# Alternative splicing reprogramming in fungal pathogen *Sclerotinia sclerotiorum* at different infection stages on *Brassica napus*

**DOI:** 10.3389/fpls.2022.1008665

**Published:** 2022-10-12

**Authors:** Xiaohui Cheng, Chuanji Zhao, Lixia Gao, Lingyi Zeng, Yu Xu, Fan Liu, Junyan Huang, Lijiang Liu, Shengyi Liu, Xiong Zhang

**Affiliations:** ^1^ Key Laboratory of Biology and Genetic Improvement of Oil Crops, Ministry of Agriculture of the People’s Republic of China (PRC), Oil Crops Research Institute, Chinese Academy of Agricultural Sciences, Wuhan, China; ^2^ State Key Laboratory of Agricultural Microbiology, Huazhong Agricultural University, Wuhan, China; ^3^ Hebei Provincial Academy of Ecological and Environmental Sciences, Shijiazhuang, China

**Keywords:** *Sclerotinia sclerotiorum*, transcriptome sequencing, alternative splicing, reprogramming, secreted proteins

## Abstract

Alternative splicing (AS) is an important post-transcriptional mechanism promoting the diversity of transcripts and proteins to regulate various life processes in eukaryotes. Sclerotinia stem rot is a major disease of *Brassica napus* caused by Sclerotinia sclerotiorum, which causes severe yield loss in *B. napus* production worldwide. Although many transcriptome studies have been carried out on the growth, development, and infection of S. sclerotiorum, the genome-wide AS events of S. sclerotiorum remain poorly understood, particularly at the infection stage. In this study, transcriptome sequencing was performed to systematically explore the genome-scale AS events of *S. sclerotiorum* at five important infection stages on a susceptible oilseed rape cultivar. A total of 130 genes were predicted to be involved in AS from the *S. sclerotiorum* genome, among which 98 genes were differentially expressed and may be responsible for AS reprogramming for its successful infection. In addition, 641 differential alternative splicing genes (DASGs) were identified during *S. sclerotiorum* infection, accounting for 5.76% of all annotated *S. sclerotiorum* genes, and 71 DASGs were commonly found at all the five infection stages. The most dominant AS type of *S. sclerotiorum* was found to be retained introns or alternative 3′ splice sites. Furthermore, the resultant AS isoforms of 21 DASGs became pseudogenes, and 60 DASGs encoded different putative proteins with different domains. More importantly, 16 DASGs of *S. sclerotiorum* were found to have signal peptides and possibly encode putative effectors to facilitate the infection of *S. sclerotiorum*. Finally, about 69.27% of DASGs were found to be non-differentially expressed genes, indicating that AS serves as another important way to regulate the infection of *S. sclerotiorum* on plants besides the gene expression level. Taken together, this study provides a genome-wide landscape for the AS of *S. sclerotiorum* during infection as well as an important resource for further elucidating the pathogenic mechanisms of *S. sclerotiorum*.

## Introduction

Alternative splicing (AS) of precursor mRNAs is an important mechanism to increase transcriptome and proteome diversity through the production of multiple mRNA isoforms from a single coding gene (Chaudhary et al., 2019). The splicing is catalyzed by two unique spliceosomes designated as major U2-dependent spliceosome and minor U12-dependent spliceosome. Notably, the U2-dependent spliceosome mainly processes GT-AG introns, while the U12-dependent spliceosome processes AT-AC introns (Turunen et al., 2013). AS events can be classified into five major types: alternative 3′ splice site (A3SS), alternative 5′ splice site (A5SS), exon skipping (ES), retained intron (RI), and mutually exclusive exon (MXE). Specifically, RI is the most frequent type of AS in plants (Reddy et al., 2013), whereas ES is the most frequent type in animals (Pan et al., 2009). About 95% of human and 60% of *Arabidopsis* genes undergo AS events during expression, and many of these genes can generate multiple splicing isoforms (Pan et al., 2009; Zhang et al., 2017a)—for example, the small-sized *Arabidopsis* genome encodes 82,190 nonredundant isoforms from 34,212 genes (Zhang et al., 2017a). The most extreme example of AS is the *Drosophila melanogaster* gene *DSCAM*, which potentially encodes 38,016 distinct isoforms through AS (Graveley, 2005). AS has likewise been reported in microbial eukaryotes, and the ratio ranges from 0.2% in the non-pathogenic yeast *Saccharomyces cerevisiae* (Grutzmann et al., 2014) to 24% in the plant-pathogenic oomycete *Pseudoperonospora cubensis* (Burkhardt et al., 2015), which is much lower than that in plants and animals.

As a major posttranscriptional regulatory event of eukaryotes, AS plays essential roles in various cellular functions and adaptation to environmental changes. In humans, aberrant AS is associated with several diseases, such as cancer (Bonnal et al., 2020), muscular dystrophies (Pistoni et al., 2010), and neurodegenerative diseases (Tollervey et al., 2011). In plants, AS is important for growth and development (Wang et al., 2020), circadian clock (James et al., 2012), stress response (Palusa and Reddy, 2015), and immune response (Huang et al., 2020). Despite the great progress in related research, AS events and regulation in microbial eukaryotes remain largely elusive, especially in plant pathogens. A genome-wide study of 23 fungal genomes has revealed that pathogenic species have much higher AS rates than non-pathogenic species (Grutzmann et al., 2014). Host-specific and infection-specific AS were observed in *S. sclerotiorum* and *Magnaporthe oryzae* (Ibrahim et al., 2021; Jeon et al., 2022), respectively. In *Ustilago maydis*, the two core glycolysis enzymes, GAPDH and PGK, can generate two isoforms through AS, one of which is located on the peroxisome, and the deletion of the peroxisome-located isoform would reduce its virulence to maize (Freitag et al., 2012). Intriguingly, signal peptide-containing proteins and putative effectors were observed to undergo AS in *P. cubensis* (Burkhardt et al., 2015). Based on these findings, it can be speculated that AS is functionally regulated during the host colonization of plant pathogens.

*S. sclerotiorum* is a notorious pathogen infecting more than 400 host plants all over the world, including some economically important crops, such as tomato (*Solanum lycopersicum)*, common bean (*Phaseolus vulgaris*), sunflower (*Helianthus annuus*), and oilseed rape (*Brassica napus* L.) (Bolton et al., 2006). Sclerotinia stem rot caused by *S. sclerotiorum* is a major yield-limiting factor, causing about $200 million of annual economic loss in the United States alone (Bolton et al., 2006). For successful infection, *S. sclerotiorum* has evolved intricate strategies to disrupt host recognition (Kabbage et al., 2015). Among these strategies, oxalic acid has attracted great research attention since it manipulates the host redox environment and pH signaling to promote pathogenicity during *S. sclerotiorum* disease development (Williams et al., 2011). In addition, some effectors, such as an integrin-like protein SsITL (Zhu et al., 2013), a putative Ca^2+^ binding protein Ss-caF1 (Xiao et al., 2014), an uncharacterized protein SsSSVP1 (Lyu et al., 2016), and a cerato-platanin protein SsCP1 (Yang et al., 2018), are commonly involved in the virulence of *S. sclerotiorum*. Overall, most studies of *S. sclerotiorum* pathogenicity have been mainly focused on oxalic acid, effectors, transcription factors, and cell-wall-degrading enzymes (Xu et al., 2018). Although there have been increasing transcriptomic data to determine the gene expression profiles of *S. sclerotiorum* (Peng et al., 2017; Westrick et al., 2019; Chittem et al., 2020; Xu et al., 2021), the prevalence of genome-wide AS in *S. sclerotiorum* at different infection stages remains elusive.

Clarification of how this pathogen infects hosts will improve our understanding of *S. sclerotiorum* and facilitate the development of disease-resistant crops. High-throughput transcriptome profiling is a powerful approach for the quantitative profiling of AS (Wang et al., 2008; Wang et al., 2009). However, AS events across different infection stages are still largely unknown. Although host-specific AS has been characterized in *S. sclerotiorum*, the study was based on samples collected at only one infection stage. In the present study, we used RNA-seq technology to detect the genome-wide AS events of *S. sclerotiorum* across different stages of infection, including mycelia (MY) and at 2, 6, 12, 24, and 36 h post-inoculation (hpi) on susceptible oilseed rape. The AS characteristics in *S. sclerotiorum* were elucidated, and more importantly, the effects of AS on the function of proteins were comprehensively analyzed, particularly secreted proteins. The findings provide a comprehensive landscape of AS during *S. sclerotiorum* infection and important gene resources for future research on its pathogenic mechanisms.

## Materials and methods

### RNA-seq sample collection

The S. *sclerotiorum* strain 1980 was collected from oilseed rape stems in the field and grown on potato dextrose agar (PDA) to produce a hyphal inoculum. Susceptible oilseed rape (*Brassica napus* cv Westar) was used as the host in this study. The plants were grown in a growth chamber at 25°C, 80% humidity, and 14-h light/8-h dark cycle. Once germinated, the seedlings were watered when necessary and fertilized once a week with 20–20–20 fertilizer. At 4weeks after planting, the plants were infected with *S. sclerotiorum.*

Mycelia were collected from the cellophane placed on the PDA immediately prior to inoculation (time 0) and at 2, 6, 12, 24, and 36 h post-inoculation (hpi). The collected samples were immediately frozen in liquid nitrogen and stored at −80°C for RNA extraction.

### RNA isolation, RNA-seq library preparation, and sequencing

Total RNA was extracted from the collected samples using the EZNA total RNA kit I (Omega BioTek, Norcross, GA, USA) following the manufacturer’s instructions. RNA quality was checked using a Qubit fluorometer (Life Technologies, New York, NY, USA) and Bioanalyzer 2100 (Agilent Technologies, Santa Clara, CA, USA). The libraries were then constructed and sequenced on Illumina HiSeq X Ten using the paired-end mode (Novogene Co. Ltd., Beijing, China). Three biological replicates were used for each analysis. All raw sequence data generated in this study have been deposited in the NCBI Sequence Read Archive (available online) under Bioproject ID PRJNA777355.

### Transcriptomic analysis

Adapter trimming and low-quality read filtering were carried out by fastp (Chen et al., 2018) with default settings. The *S. sclerotiorum* near complete reference genome sequence (Derbyshire et al., 2017) and gene model annotation files were retrieved from the FTP site provided by NCBI. The paired-end clean reads were mapped to the reference genome by the software TopHat v2.0.12 (Trapnell et al., 2009) using the following option: -an 8 -m 0 with an anchor length of more than 8 nt for the spliced alignments. The gene expression levels were quantified by fragments per kilobase of transcript sequence per million base pairs mapped reads (FPKM) (Trapnell et al., 2010). The similarity between samples at the expression level was estimated by calculating the Pearson correlation coefficient and then visualized using R package pheatmap. Differentially expressed genes (DEGs) were determined by the R package DESeq (Anders and Huber, 2010) with an adjusted *P*-value <0.05 (false discovery rate = 0.05), and the absolute value of the log_2_ fold change ratio was >2. Fold change values were the ratio of FPKM of the different samples to that of the control.

### Identification of differential alternative splicing

All putative AS events were extracted from the abovementioned high-quality transcript GTF file using rMATS (Shen et al., 2014). Five major types of AS were detected, including A5SS, A3SS, ES, IR, and MXE. The events with a *P*-value <0.05 calculated by rMATS were identified as significantly differential events. The Sashimi plot was constructed using rmats2sashimiplot (https://github.com/Xinglab/rmats2sashimiplot).

### Gene function analysis

For secretome annotation, SignalP 3.0 (Bendtsen et al., 2004) and TMHMM v. 2.0 (Krogh et al., 2001) software were used to predict the presence of signal peptides and transmembrane (TM) domains, respectively. Proteins with signal peptides but no TM domains were considered as candidate secreted proteins. *S. sclerotiorum* gene ontology (GO) terms were obtained from a previous study (Derbyshire et al., 2017). GO enrichment analysis was performed using the OmicShare tools, a free online platform for data analysis (www.omicshare.com/tools). A protein–protein interaction network was constructed using the STRING database (http://string-db.org) with a combined score of >0.4. Sources in STRING include experimentally determined interactions, curated databases, and information of neighborhood, co-occurrence, text mining, co-expression, and homology (Szklarczyk et al., 2021).

### qRT-PCR validation of candidate genes

Total RNA samples were isolated at different infection stages. First-strand cDNA was obtained with the Prime Script RT reagent kit (Takara Bio, Beijing, China), and then real-time PCR was run on the CFX Connect Real-time PCR system (Bio-Rad, Hercules, CA, USA). The *S. sclerotiorum* actin gene (*Sscle_14g099090*) was used to normalize the gene expression. Three biological replicates were performed per sample, and the relative gene expression levels were calculated using the 2^−ΔΔCt^ method (Livak and Schmittgen, 2001). The primers are listed in **Supplementary Table S1**.

## Results

### Time-course transcriptome of *S. sclerotiorum* infection on *B. napus*

To characterize the transcriptome and AS patterns of *S. sclerotiorum* at different infection stages on *B. napus*, RNA-seq of *S. sclerotiorum* was performed at 0 (mycelia), 2, 6, 12, 24, and 36 hpi with three biological replicates. After data trimming and quality filtering, a total of 354.78 million clean paired-end reads were obtained for 18 samples, with the reads for each sample ranging from 16.14 to 28.57 million (**Supplementary Table S2**). Approximately 83.56% of the clean reads were mapped to the *S. sclerotiorum* 1980 UF-70 reference genome (Derbyshire et al., 2017). As a result, among the 11,130 protein-coding genes (Derbyshire et al., 2017), 9,512 genes were defined as expressed genes (FPKM ≥1.0) in at least one sample, indicating that the RNA-seq analysis had sufficient coverage.

**Figure 1:**
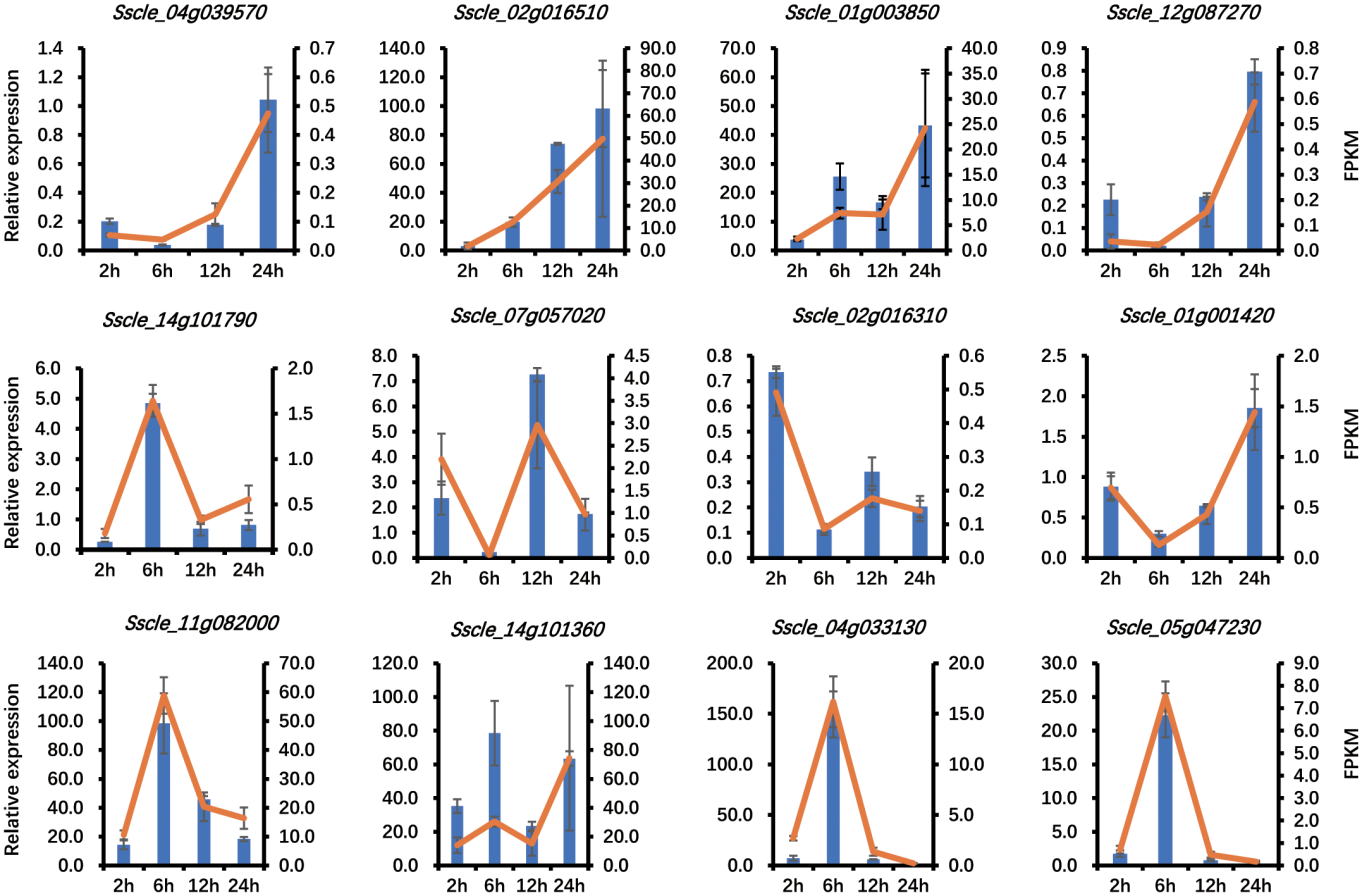
RNA-seq data validation by quantitative real-time PCR (qRT-PCR). The qRT-PCR results are presented as relative expression using actin as a reference gene. The expression levels are presented as the mean of three biological replicates, and the error bars show the standard deviation.

To confirm the quality of the transcriptome generated in this study, Pearson correlation coefficient (*r* ^2^) was calculated between biological replicates based on the FPKM values of all the detected genes. Hierarchical clustering analysis showed that the data of three replicates were tightly clustered together, and independent treatments were clearly separated from each other (**Supplementary Figure S1A**), indicating the reliability of the experiments. The separation was even more evident in the principal component analysis (**Supplementary Figure S1B**). To further test the reliability of the RNA-seq results from Illumina sequencing data, 12 genes were randomly selected to carry out quantitative real-time PCR (qRT-PCR) validation, and the yielded results were consistent with our RNA-seq data (**Figure 1**). These results further confirmed that our RNA-seq data are accurate and reliable.

### Splicing-related genes are active at different infection stages

**Figure 2:**
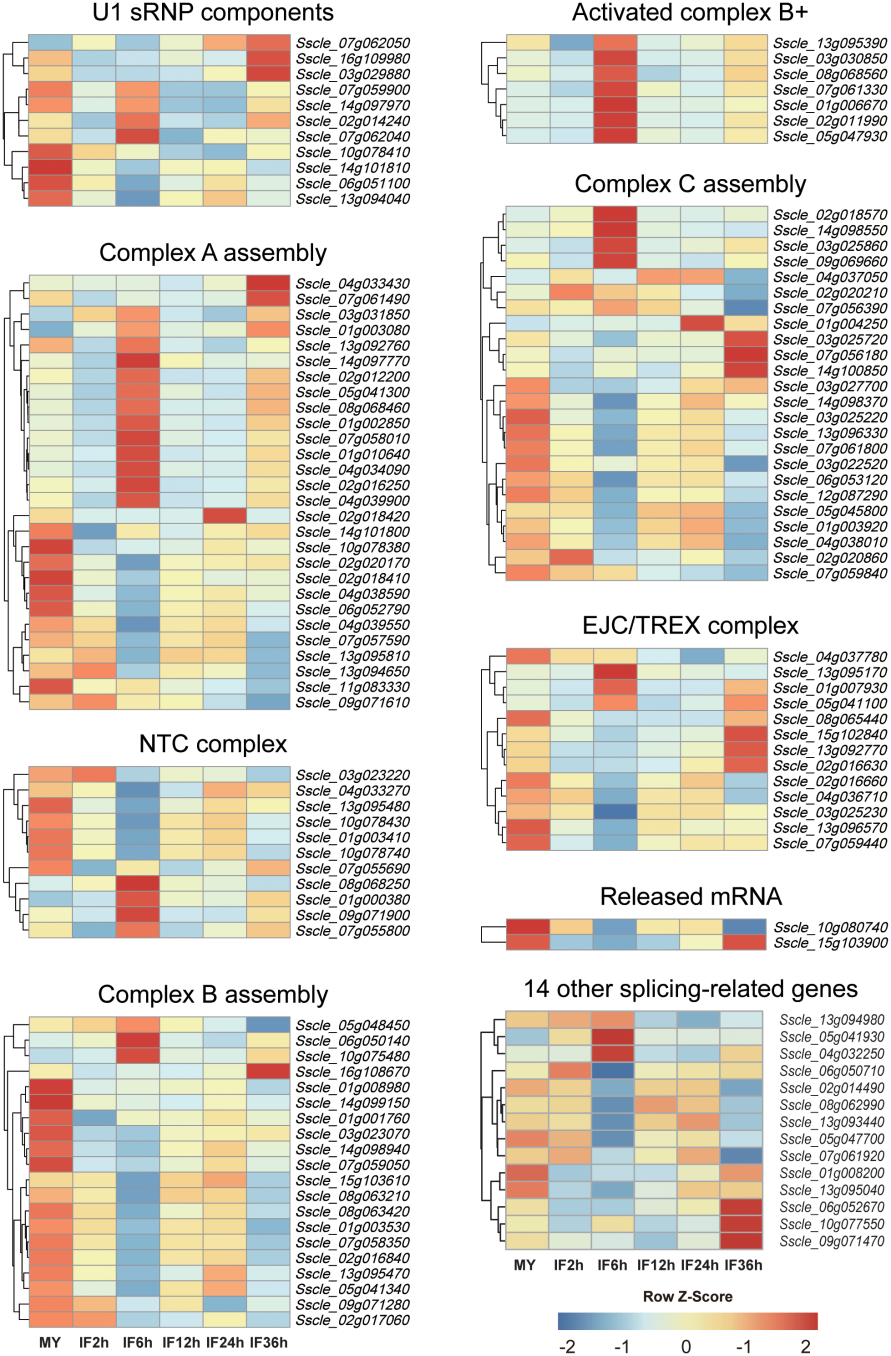
Expression patterns of splicing‐related genes during *S. sclerotiorum* infection on *Brassica napus*. The gene ID of splicing factors is listed in the heat map. Blue to red indicate gene expression values from low to high. *Z* score normalization was applied for the transcription levels of genes at all stages. Cluster analysis was performed using the R package pheatmap.

Pre-mRNA splicing is catalyzed by a higher-order protein complex termed as the spliceosome, which comprises a range of proteins called splicing‐related proteins (Will and Luhrmann, 2011; Matera and Wang, 2014). The abundance and the activity of splicing-related genes govern the AS patterns of target genes and their expression patterns under different environmental conditions (Syed et al., 2012; Papasaikas et al., 2015). To gain more insights into the genome-wide AS of *S. sclerotiorum* at different infection stages, 116 potential splicing-related genes previously identified in *S. sclerotiorum* genome (Ibrahim et al., 2021) were subjected to expression analysis (**Figure 2** and **Supplementary Table S3**). As a result, all genes were expressed at detectable levels (FPKM ≥1.0) except for *Sscle02g018420*, which encodes a U2AF, and 105 genes showed high expression (FPKM ≥20.0) in at least one infection stage (**Figure 2**). Importantly, among the 116 splicing-related genes, 93 genes were differentially expressed at the five infection stages compared with the mycelium stage, including 31 upregulated genes and 65 downregulated genes—for example, *Sscle_01g006670*, which encodes an LSM2 homolog, was upregulated by 8.3 folds at 36 hpi, while the RNA helicase p68-coding gene *Sscle_03g029880* displayed 53.6-fold downregulation at 6 hpi. Interestingly, *Sscle_16g108670*, which encodes a Snu13 homolog, was dramatically downregulated at 2 hpi but upregulated at 36 hpi. Moreover, the SMG1-coding gene *Sscle_05g041300* and the SmD1-coding gene *Sscle_04g039900* were both downregulated at 2 hpi but upregulated at 6 hpi.

Additionally, we identified 14 other splicing-related genes based on the GO terms. Similarly, these genes also exhibited high expression levels at all infection stages, and there were five DEGs (one upregulated and four downregulated) relative to the mycelium stage (**Figure 2** and **Supplementary Table S3**)—for example, *Sscle_01g008200* was dramatically downregulated at 2, 6, 12, and 24 hpi, while *Sscle_05g041930* showed an increase in expression at 6 hpi. Overall, these results demonstrated that *S. sclerotiorum* infection has significant effects on spliceosome components.

### Prevalent changes in mRNA alternative splicing are observed in *S. sclerotiorum* during infection

**Figure 3:**
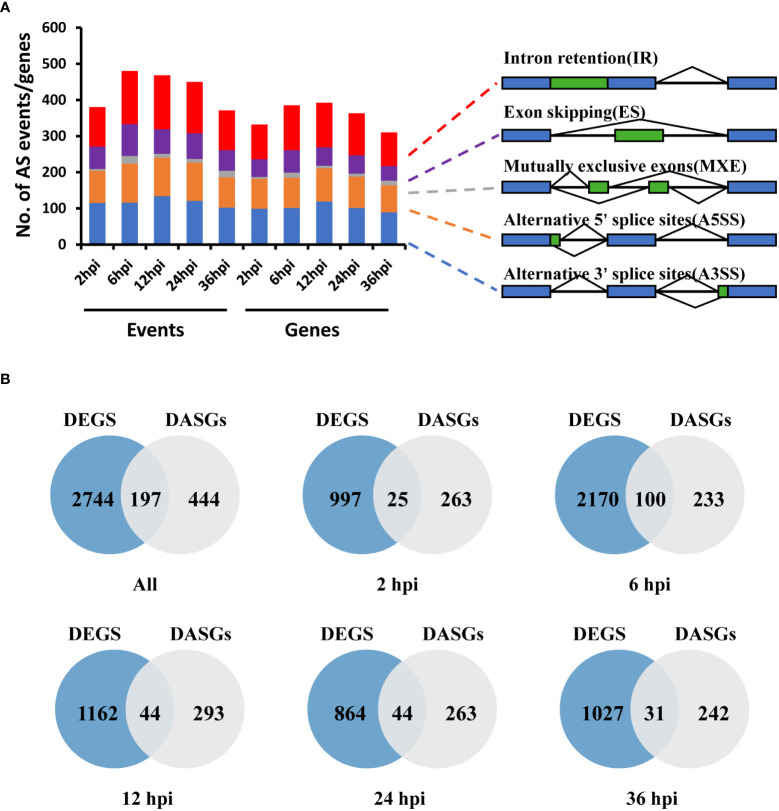
Differential alternative splicing genes (DASGs) and differentially expressed genes (DEGs) in *S. sclerotiorum* at different infection stages. **(A)** Number of different types of differentially expressing events and corresponding genes identified in different infected samples. A3SS, alternative 3′ splice site; A5SS, alternative 5′ splice site; ES, exon skipping; RI, retained intron; MXE, mutually exclusive exon. **(B)** Venn diagram of the overlap of DEGs and DASGs.

To determine the dynamic changes of AS in *S. sclerotiorum* pre-mRNA during infection on *B. napus*, we detected the host-induced differential alternative splicing (DAS) events in *S. sclerotiorum* by comparing the infection samples with the mycelium sample from the RNA-seq data. The number of DAS events ranged from 371 at 36 hpi to 480 at 6 hpi, and there was a total of 2,149 DAS events covering 641 gene loci (**Figure 3A**). Among these genes, 71 genes were found in all infection stages (**Supplementary Figure S2A**), and each DAS gene corresponded to an average of 3.35 DAS events. These DAS genes (DASGs) accounted for 6.74% of the 9,512 genes expressed in *S. sclerotiorum* genome. In terms of the distribution of different types of AS, retained intron (RI; 30.47 ± 1.32%) was the most detected, followed by alternative 3′ splice site (A3SS; 27.49 ± 2.26%), alternative 5′ splice site (A5SS; 22.95 ± 0.49%), exon skipping (ES; 16.06 ± 1.43%), and mutually exclusive exon (MXE; 3.02 ± 1.51%). Most genes had a single type of DAS, and there were 117 genes exhibiting multiple types of DAS (**Supplementary Figure S2B**).

To investigate the regulatory mechanism of AS during infection, a pairwise comparison of DEGs and DASGs was further performed. A total of 2,941 DEGs were detected, including 1,022, 2,270, 1,206, 908, and 1,058 DEGs at 2, 6, 12, 24, and 36 hpi, respectively (**Figure 3B**). The results showed that the gene overlapping rate between all DEGs and DASGs was as low as 5.82%. Specifically, the number of overlapping genes was 25 for 2 hpi, 100 for 6 hpi, 44 for 12 hpi, 44 for 24 hpi, and 31 for 36 hpi, whereas 263, 233, 293, 263, and 242 DASGs were not differentially expressed at 2, 6, 12, 24, and 36 hpi, respectively (**Figure 3B**). The fact that most DASGs were not differentially expressed at different infection stages indicated that AS is independent of gene expression regulation during the infection process of *S. sclerotiorum*. Indeed the GO enrichment analysis revealed that there were significant differences in the biological process between DASGs and DEGs. As shown in **Table 1** and **Supplementary Figure S3**, DASGs were enriched in processes such as the cellular modified amino acid metabolic process (GO:0006575), organonitrogen compound catabolic process (GO:1901565), and heterocycle biosynthetic process (GO:0018130), while DEGs were enriched in ribosome biogenesis (GO:0042254), ribonucleoprotein complex biogenesis (GO:0022613), and rRNA processing (GO:0006364).

**Table 1:** Top 10 biological processes from the Gene Ontology (GO) enrichment analysis of differentially expressed genes (DEGs) and differential alternative splicing genes (DASGs).

GO description (DEGs)	*P*-value	GO description (DASGs)	*P*-value
Ribosome biogenesis	7.57E-25	Cellular modified amino acid metabolic process	7.71E-05
Ribonucleoprotein complex biogenesis	1.32E-21	Organonitrogen compound catabolic process	1.01E-04
rRNA processing	1.08E-17	Heterocycle biosynthetic process	2.42E-04
rRNA metabolic process	1.08E-17	Nucleobase-containing compound biosynthetic	3.50E-04
ncRNA processing	1.62E-11	Kynurenine metabolic process	4.02E-04
Carbohydrate metabolic process	4.68E-09	Aromatic compound biosynthetic process	5.36E-04
ncRNA metabolic process	1.38E-08	Tryptophan catabolic process	1.12E-03
Cellular component biogenesis	1.28E-06	Amine catabolic process	1.12E-03
Polysaccharide catabolic process	3.64E-06	Cellular biogenic amine catabolic process	1.12E-03
Polysaccharide metabolic process	7.84E-06	Indole-containing compound catabolic process	1.12E-03

### Alternative splicing variants with modified functions

To evaluate the potential impacts of AS on protein function, we examined the transcript isoforms of genes resulting from AS events and the resultant protein sequences. As a result, the AS isoforms of 21 genes were found to be truncated in the open reading frames to less than 30 amino acids, and therefore these alternative isoforms should be pseudogenized (**Table 2**). Among these 21 genes, a *Fusarium graminearum* pre-mRNA-splicing factor srp1 (Zhang et al., 2017b) homolog-coding gene *Sscle_09g071470*, a myb-like domain-containing protein-coding gene *Sscle_05g047440*, and an enolase-phosphatase E1-coding gene *Sscle_03g030320* were found at all infection stages, while 11 genes were detected at only one infection stage, and eight genes were found in two to four infection stages. We then found that all AS types led to pseudogenization except for MXE—for example, the A3SS, A5SS, SE, and RI types of AS led to the pseudogenization of a dynactin subunit-coding gene *Sscle_03g022490*, a thiamine thiazole synthase-coding gene *Sscle_01g000960*, a calmodulin-1-coding gene Sscle_01g010310, and an ACT_7 domain-containing protein coding gene *Sscle_01g007440*, respectively (**Supplementary Figure S4**).

**Table 2:** Differential alternative splicing genes with pseudogenization.

Gene ID	AS type	Infection stage	Description
Sscle_07g059110	RI	2 hpi	IMP-specific 5′-nucleotidase
Sscle_12g091700	A5SS	2 hpi	Alkaline ceramidase
Sscle_01g010310	SE	2 hpi	Calmodulin-1
Sscle_08g065510	A5SS	6 hpi	GH16 domain-containing protein
Sscle_03g022490	A3SS	6 hpi	Dynactin subunit
Sscle_04g033380	RI	6 hpi	Ubiquitin-conjugating enzyme E2
Sscle_04g037580	SE	6 hpi	Uncharacterized protein
Sscle_05g045150	A3SS	6 hpi	Tyrosine-protein phosphatase
Sscle_08g066420	SE	12 hpi	C2H2-type domain-containing protein
Sscle_04g032930	SE	12 hpi	Ubiquitin fusion degradation protein
Sscle_01g000960	A5SS	36 hpi	Thiamine thiazole synthase
Sscle_01g003590	SE	6/36 hpi	Ras-related protein
Sscle_01g006710	SE	2/6/24 hpi	RNA polymerase III subunit
Sscle_07g055740	RI	6/12/24 hpi	Defective in cullin neddylation protein
Sscle_06g048720	A3SS	2/12/24 hpi	D-/L-hydantoinase subunit
Sscle_01g007440	RI	2/6/12/24 hpi	ACT_7 domain-containing protein
Sscle_02g021320	RI	6/12/24/36 hpi	Transcription factor kapC
Sscle_09g071090	A5SS	2/6/12/24 hpi	Uncharacterized protein
Sscle_09g071470	RI	All stages	Pre-mRNA-splicing factor srp1
Sscle_05g047440	SE	All stages	Myb-like domain-containing protein
Sscle_03g030320	SE	All stages	Enolase-phosphatase E1

**Figure 4:**
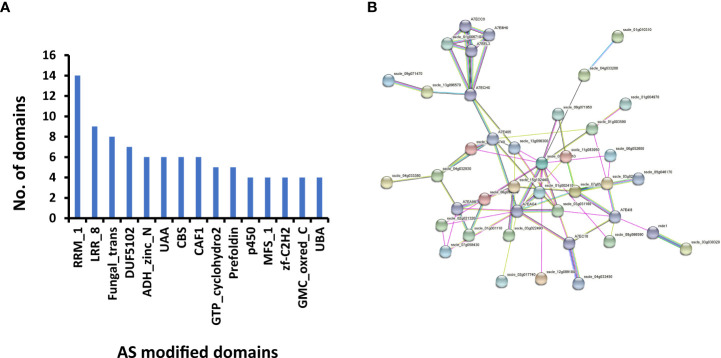
Differential alternative splicing genes (DASGs) with modified functions in *S. sclerotiorum*. **(A)** Fifteen most frequently modified domains in DASGs. **(B)** The network was constructed using STRING (https://string-db.org/). There are 41 protein nodes and 73 protein–protein association edges in the network. Seven differently colored lines represent the types of evidence used in predicting associations. Light blue line, database evidence; purple line, experimental evidence; green line, neighborhood evidence; blue line, co-occurrence evidence; yellow line, text mining evidence; black line, co-expression evidence; and dark blue lines, protein homology evidence.

To determine whether there are changes in the domain structures of protein isoforms resulting from AS, we predicted the domain structures of all AS isoforms. In total, 60 DASGs were found to have variations in protein domain structure. Among the proteins encoded by these DASGs, 48 protein isoforms exhibited a loss of domains, seven showed a gain of domains, and six displayed more complex changes in domains. RRM_1, LRR_8, and Fungal_trans were the three most frequently modified domains (**Figure 4A**). Only three of the 60 DASGs underwent changes in domain at all five infection stages, *i*.*e*., a probable prefoldin subunit-coding gene *Sscle_13g096300*, a *F. graminearum* pre-mRNA-factor srp2 (Zhang et al., 2020b) homologous-coding gene *Sscle_13g096570*, and a D-arabinitol dehydrogenase-coding gene *Sscle_16g110540*. Sscle_13g096300, Sscle_13g096570, and Sscle_16g110540 isoforms lost the prefoldin domain, RRM_1 domain, and ADH_zinc_N domain, respectively. Both RI and SE types were found to contribute to the loss of ADH_zinc_N domain in the Sscle_16g110540 isoforms.

Furthermore, the interaction network of all 81 DASGs with pseudogenization and/or domain changes was constructed. As shown in **Figure 4B**, 31 DASGs and 10 other genes interact with each other, resulting in 73 protein–protein associations. Two proteins, Sscle_03g023610 and A7EAG4 (Sscle_04g033520), had more than 10 connections with other proteins, suggesting that they are hub genes in the network. *Sscle_03g023610* encodes a tubulin gamma chain, while *A7EAG4* encodes cell division control protein 2, which belongs to the protein kinase superfamily. In addition, we found that these two hub genes were highly expressed during infection (**Supplementary Figure S5**). Overall, the interaction network showed that part of the DASGs were biologically connected and two hub genes might play crucial roles in the infection process.

### *S. sclerotiorum* infection activates the AS of secreted proteins

**Figure 5:**
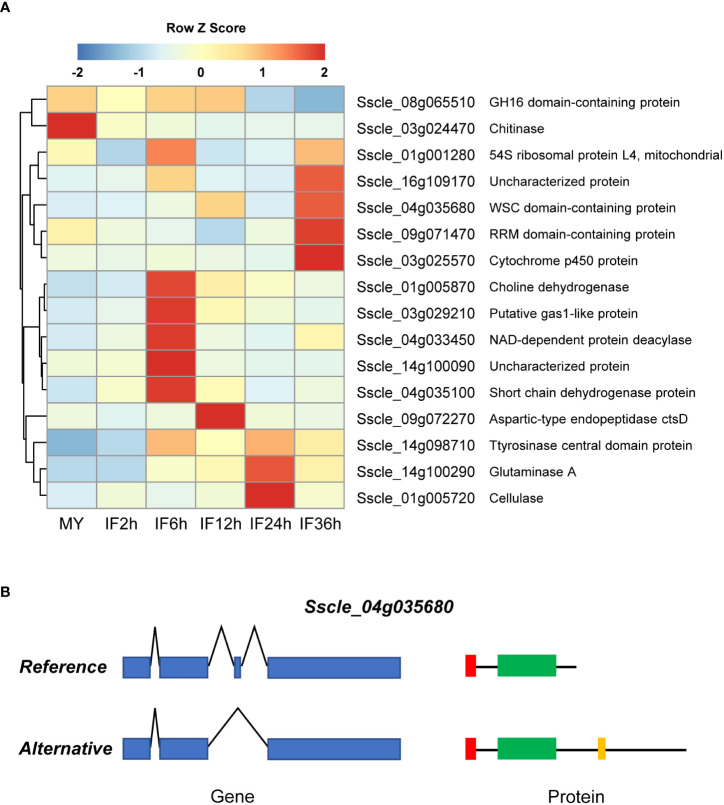
Modified functions of secreted protein-coding genes by alternative splicing (AS) in *S. sclerotiorum*. **(A)** The expression patterns of the secreted protein-coding genes undergo AS. **(B)** Schematic representation of *Sscle_04g035680* produced by AS. The blue boxes and polylines indicate the exons and introns, respectively. The red boxes, green boxes, and yellow box indicates the signal peptides, WSC domains, and transmembrane domain, respectively.

During infection and colonization, various plant pathogens target the intracellular processes of plant cells *via* deploying secreted proteins (Arroyo-Velez et al., 2020). To determine whether secreted proteins undergo AS during infection, we predicted the signal peptide and transmembrane domain of all AS isoforms. In total, 16 predicted secreted protein-coding genes were found to be alternatively spliced during infection (**Figure 5A**). The expression analysis revealed that 75.0% of secreted DASGs (12 out of 16) were significantly upregulated in at least one infection stage (**Figure 5A**), and the ratio was much higher than that of the total DASGs and secreted proteins.

Among the 16 spliced secreted proteins, two isoforms were predicted to lose the secretion function because no signal peptide (a cellulase coding gene *Sscle_01g005720* and a putative gas1-like protein coding gene *Sscle_03g029210*) was detected. One isoform was found to gain a transmembrane domain (a WSC domain-containing protein-coding gene *Sscle_04g035680*, **Figure 5B**), and two isoforms (a GH16 domain-containing protein-coding gene *Sscle_08g065510* and an RRM domain-containing protein-coding gene *Sscle_09g071470*) underwent pseudogenization. Intriguingly, there were three secreted proteins with altered domains in alternative isoforms, namely, a glutaminase-coding gene *Sscle_14g100290*, a NAD-dependent protein deacetylase-coding gene *Sscle_04g033450*, and a cytochrome p450 protein-coding gene *Sscle_03g025570*—for example, *Sscle_14g100290* encodes two secreted protein isoforms, namely, a reference protein Sscle_14g100290.1 and an alternative protein Sscle_14g100290.2: the former contained a signal peptide and three domains of unknown function (DUF4965, DUF5127, and DUF1793), while the latter contained a signal peptide and a domain of unknown function (DUF5127). In addition, loss of signal peptides and changes in domains were not observed in the alternative isoforms of the other nine secreted proteins.

## Discussion

Plant diseases pose serious impacts on global agriculture, causing significant losses in crop productivity and great threats to global food security. As a filamentous ascomycete fungus, *S. sclerotiorum* causes a devastating disease for many important crops worldwide, particularly *B. napus* (Bolton et al., 2006). Therefore, a better understanding of the pathogenic mechanisms during *S. sclerotiorum* infection is of great importance to both basic and applied research on the development of effective strategies for disease resistance in breeding. Although the gene profiles in *S. sclerotiorum* have been extensively reported and well characterized (Peng et al., 2017; Westrick et al., 2019; Chittem et al., 2020; Xu et al., 2021), gene regulation at the post-transcriptional level, such as alternative splicing across all stages of infection, remains poorly understood. In the present study, we systematically characterized the genome-wide AS in *S. sclerotiorum* at five infection stages on *B. napus*.

Genome-wide AS events have been extensively investigated for plants and animals in their response to various biotic and abiotic stresses (Reddy et al., 2013). Only a few genome-wide AS events have been identified in fungi. It has been reported that the ratio of AS events ranges from 0.2% in the non-pathogenic *Saccharomyces cerevisiae* to 9.5% in the filamentous fungus *Aspergillus niger* (Grutzmann et al., 2014) and from 2.3 to 18.2% in human pathogenic fungi (Stepankiw et al., 2015). A recent study showed that 1,487 *S. sclerotiorum* genes exhibited alternative isoforms on diverse host plants, which may contribute to expanding the host spectrum of *S. sclerotiorum* (Ibrahim et al., 2021). However, genome-wide AS across all stages of fungi infection is largely unknown, which can reflect how AS is regulated during pathogenesis. In this study, we found 2,149 AS events among 641 genes in *S. sclerotiorum*, accounting for 5.8% of all the annotated genes and suggesting a genome-wide regulation of AS process during the colonization of *S. sclerotiorum* in the host plant. Among the 641 differential alternative splicing genes identified at five post-infection time points, only 71 DASGs were shared by all five time points, indicating dramatic changes in AS during *S. sclerotiorum* infection. In terms of the different types of AS events in *S. sclerotiorum*, the most common AS type was retained intron (30.47 ± 1.32%), which is consistent with the findings in a previous study (Ibrahim et al., 2021) and in plants (Reddy et al., 2013). However, in mammals, exon skipping is the dominant AS event (Pan et al., 2009). Thus, further research should be conducted on the post-transcriptional regulation of host-induced DASGs in a plant–pathogen interaction.

Previous studies have demonstrated that gene expression comprises both the gene expression level and AS, which are independent layers in some species (Huang et al., 2019; Huang et al., 2020). To better understand the transcriptomic dynamics of AS at different infection stages, we also investigated the DEGs in *S. sclerotiorum*. A comparison between DASGs and DEGs revealed that there were only 197 common genes in these two gene sets. A further GO enrichment analysis revealed that DASGs are mainly involved in the cellular modified amino acid metabolic process, organonitrogen compound catabolic process, and heterocycle biosynthetic process. By contrast, DEGs are associated with ribosome biogenesis, ribonucleoprotein complex biogenesis, and rRNA processing. These results strongly suggest that AS can serve as an independent mechanism for gene regulation in *S. sclerotiorum* infection on *B. napus*.

A crucial issue to be addressed in this study is how S. *sclerotiorum* infection shapes AS. Numerous studies have demonstrated that AS events are directed by splicing factors and regulators in plants and animals—for example, the SR proteins in *Arabidopsis* have been shown to significantly influence the AS efficiency of their own pre-mRNAs and several other genes (Yan et al., 2017). Notably, some splicing factors have been described to be involved in the pathogenicity of fungi. In *F. graminearum*, the splicing factors FgSRP1 (Zhang et al., 2017b), FgSRP2 (Zhang et al., 2020b), and FgSRK1 (Wang et al., 2018) are essential for the infection process and AS process. In this study, we examined the changes in the transcript levels and alternatively spliced variants of S. *sclerotiorum* splicing-related genes. A total of 130 genes annotated as splicing-related factors or regulators were obtained. Most of these genes are highly expressed during infection, among which 98 are differentially expressed. Moreover, these spliceosome components showed a dynamic expression during infection, particularly at the early stage of infection, possibly to evade plant defense and establish infection. This regulation indicates a potentially strong defense pressure on *S. sclerotiorum* during early infection on *B. napus*. In addition, two predicted splicing factors Sscle_09g071470 and Sscle_13g096570, which are homologs of *F. graminearum* FgSRP1 and FgSRP2, respectively, undergo AS at all infection stages, indicating that they are promising targets for future research.

AS can expand proteome diversity and plays a crucial role in the evolution of species (Blencowe, 2006). This study is focused on the analysis of DASGs with pseudogenization, domain changes, and secretion. The results demonstrate that 21 genes are pseudogenized in alternative isoforms, which might be degraded through the nonsense-mediated decay pathway (Nickless et al., 2017). In terms of DASGs with changes in domains, RRM_1 and LRR_8 were the two most frequently modified domains. RRM_1 domain is known to bind with single-stranded RNAs, and proteins containing this domain have critical functions in regulating gene expression in eukaryotic cells, such as AS, mRNA transport, RNA editing, and modulation of mRNA translation (Zhang et al., 2016). VdNop12, a protein containing the RRM_1 domain, is a crucial factor of pathogenicity and cold adaption in *Verticillium dahliae* (Zhang et al., 2020a). LRR-containing proteins have been reported to be essential in the development and pathogenicity of *Phytophthora sojae* (Si et al., 2021). Two hub genes were identified in the protein–protein interaction network, which may be involved in regulating the pathogenicity of *S. sclerotiorum*. More importantly, 16 spliced secreted proteins were identified in this study. Secreted effectors are key virulence determinants in plant pathogens (Arroyo-Velez et al., 2020). Thus, we believe that AS reprogramming confers fitness benefits to *S. sclerotiorum* at different infection stages, and these secreted proteins undergoing AS are of great significance for the infection of *S. sclerotiorum*.

In this study, we performed the genome-wide AS analysis of *S. sclerotiorum* across all stages of infection. Our results demonstrate that AS is prevalent in *S. sclerotiorum*. In addition, we found that AS can give rise to functionally divergent proteins, and *S. sclerotiorum* infection activates the AS of secreted proteins. Taken together, our findings shed a new light on the landscape of AS and its potential contribution to *S. sclerotiorum* infection.

## Data availability statement

The datasets presented in this study can be found in online repositories. The names of the repository/repositories and accession number(s) can be found in the article/**Supplementary Material**.

## Author contributions

XZ and SL directed the project. XC, CZ, LG, YX, and LL analyzed the data. LZ, LF, JH, and XZ performed the experiments. XC and XZ wrote the manuscript. All authors contributed to the article and approved the submitted version.

## Funding

The project was supported by the Central Public-Interest Scientific Institution Basal Research Fund (Y2020YJ03 and 1610172021001), the China Agriculture Research System of MOF and MARA (CAAS-12), and the Agricultural Science and Technology Innovation Program of the Chinese Academy of Agricultural Sciences (CAAS-ASTIP-2013-OCRI and CAAS-OCRI-XKPY-202104).

## Conflict of interest

The authors declare that the research was conducted in the absence of any commercial or financial relationships that could be construed as a potential conflict of interest.

## Publisher’s note

All claims expressed in this article are solely those of the authors and do not necessarily represent those of their affiliated organizations, or those of the publisher, the editors and the reviewers. Any product that may be evaluated in this article, or claim that may be made by its manufacturer, is not guaranteed or endorsed by the publisher.

## Supplementary material

Supplementary Figure 1 Correlation between the RNA-seq data. **(A)** Hierarchical clustering analysis of the gene expression levels in *S. sclerotiorum* at 0, 2, 6, 12, 24, and 36 hpi. The color bar represents the distance between samples, with red signifying a short distance and blue signifying a long distance between samples. The color bar (from blue to red) refers to the distance metric used for clustering, with red indicating the maximum correlation value and blue representing the minimum correlation value. **(B)** Principal component analysis of the gene expression levels in *S. sclerotiorum* at different infection stages. Each treatment has three independent biological replicates.

Supplementary Figure 2 Venn diagram of differential alternative splicing genes (DASGs). **(A)** Venn diagram of DASGs at different infection stages. **(B)** Venn diagram of DASGs under five alternative splicing types. A3SS, alternative 3′ splice site; A5SS, alternative 5′ splice site; ES, exon skipping; RI, retained intron; MXE, mutually exclusive exon.

Supplementary Figure 3 Top 20 Gene Ontology (GO) enrichment biological processes of differentially expressed genes and differential alternative splicing genes. The *x*-axis represents the negative log_10_ of the *P*-values for each GO term, and the *y*-axis indicates the GO terms.

Supplementary Figure 4 Sashimi plots for differential alternative splicing genes (DASGs) in each alternative splicing type. The plots indicate the average RNA-seq read density and splice junction counts for each DASG. The *x*-axis indicates genomic coordinates, and the *y*-axis indicates per-base expression. The numbers in the figure show the count of reads across this junction or in this area. The bottom track represents the exon–intron structure (exons in black and introns as lines) of alternative isoforms. **(A)** Sashimi plot of gene *Sscle_03g022490* with A3SS event. **(B)** Sashimi plot of gene *Sscle_01g000960* with A5SS event. **(C)** Sashimi plot of gene *Sscle_01g010310* with SE event. **(D)** Sashimi plot of gene *Sscle_01g007440* with RI event.

Supplementary Figure 5 Expression patterns of two hub genes in the STRING network. The *x*-axis represents the infection stages. The *y*-axis represents the fragments per kilobase of transcript sequence per million base pairs mapped reads value of the transcriptome.

## References

[B1] Anders S. Huber W. (2010). Differential expression analysis for sequence count data. Genome Biol. 11, R106. doi: 10.1186/gb-2010-11-10-r106 20979621 PMC3218662

[B2] Arroyo-Velez N. Gonzalez-Fuente M. Peeters N. Lauber E. Noel L. D. (2020). From effectors to effectomes: Are functional studies of individual effectors enough to decipher plant pathogen infectious strategies? PloS Pathog. 16, e1009059. doi: 10.1371/journal.ppat.1009059 33270803 PMC7714205

[B3] Bendtsen J. D. Nielsen H. Von Heijne G. Brunak S. (2004). Improved prediction of signal peptides: SignalP 3.0. J. Mol. Biol. 340, 783–795. doi: 10.1016/j.jmb.2004.05.028 15223320

[B4] Blencowe B. J. (2006). Alternative splicing: New insights from global analyses. Cell 126, 37–47. doi: 10.1016/j.cell.2006.06.023 16839875

[B5] Bolton M. D. Thomma B. P. H. J. Nelson B. D. (2006). Sclerotinia sclerotiorum (Lib.) de bary: biology and molecular traits of a cosmopolitan pathogen. Mol. Plant Pathol. 7, 1–16. doi: 10.1111/j.1364-3703.2005.00316.x 20507424

[B6] Bonnal S. C. Lopez-Oreja I. Valcarcel J. (2020). Roles and mechanisms of alternative splicing in cancer - implications for care. Nat. Rev. Clin. Oncol. 17, 457–474. doi: 10.1038/s41571-020-0350-x 32303702

[B7] Burkhardt A. Buchanan A. Cumbie J. S. Savory E. A. Chang J. H. Day B. (2015). Alternative splicing in the obligate biotrophic oomycete pathogen pseudoperonospora cubensis. Mol. Plant-Microbe Interact. 28, 298–309. doi: 10.1094/MPMI-09-14-0300-FI 25372122

[B8] Chaudhary S. Jabre I. Reddy A. S. N. Staiger D. Syed N. H. (2019). Perspective on alternative splicing and proteome complexity in plants. Trends Plant Sci. 24, 496–506. doi: 10.1016/j.tplants.2019.02.006 30852095

[B9] Chen S. F. Zhou Y. Q. Chen Y. R. Gu J. (2018). Fastp: an ultra-fast all-in-one FASTQ preprocessor. Bioinformatics 34, 884–890. doi: 10.1093/bioinformatics/bty560 30423086 PMC6129281

[B10] Chittem K. Yajima W. R. Goswami R. S. Mendoza L. E. D. (2020). Transcriptome analysis of the plant pathogen sclerotinia sclerotiorum interaction with resistant and susceptible canola (Brassica napus) lines. PloS One 15, e0229844. doi: 10.1371/journal.pone.0229844 32160211 PMC7065775

[B11] Derbyshire M. Denton-Giles M. Hegedus D. Seifbarghi S. Rollins J. Van Kan J.. (2017). The complete genome sequence of the phytopathogenic fungus sclerotinia sclerotiorum reveals insights into the genome architecture of broad host range pathogens. Genome Biol. Evol. 9, 593–618. doi: 10.1093/gbe/evx030 28204478 PMC5381539

[B12] Freitag J. Ast J. Bolker M. (2012). Cryptic peroxisomal targeting *via* alternative splicing and stop codon read-through in fungi. Nature 485, 522–U135. doi: 10.1038/nature11051 22622582

[B13] Graveley B. R. (2005). Mutually exclusive splicing of the insect dscam pre-mRNA directed by competing intronic RNA secondary structures. Cell 123, 65–73. doi: 10.1016/j.cell.2005.07.028 16213213 PMC2366815

[B14] Grutzmann K. Szafranski K. Pohl M. Voigt K. Petzold A. Schuster S. (2014). Fungal alternative splicing is associated with multicellular complexity and virulence: A genome-wide multi-species study. DNA Res. 21, 27–39. doi: 10.1093/dnares/dst038 24122896 PMC3925392

[B15] Huang W. Chen X. Guan Q. J. Zhong Z. H. Ma J. Yang B. X.. (2019). Changes of alternative splicing in arabidopsis thaliana grown under different CO2 concentrations. Gene 689, 43–50. doi: 10.1016/j.gene.2018.11.083 30528270

[B16] Huang J. Lu X. Y. Wu H. W. Xie Y. C. Peng Q. Gu L. F.. (2020). Phytophthora effectors modulate genome-wide alternative splicing of host mRNAs to reprogram plant immunity. Mol. Plant 13, 1470–1484. doi: 10.1016/j.molp.2020.07.007 32693165

[B17] Ibrahim H. M. M. Kusch S. Didelon M. Raffaele S. (2021). Genome-wide alternative splicing profiling in the fungal plant pathogen sclerotinia sclerotiorum during the colonization of diverse host families. Mol. Plant Pathol. 22, 31–47. doi: 10.1111/mpp.13006 33111422 PMC7749757

[B18] James A. B. Syed N. H. Bordage S. Marshall J. Nimmo G. A. Jenkins G. I.. (2012). Alternative splicing mediates responses of the arabidopsis circadian clock to temperature changes. Plant Cell 24, 961–981. doi: 10.1105/tpc.111.093948 22408072 PMC3336117

[B19] Jeon J. Kim K. T. Choi J. Cheong K. Ko J. Choi G.. (2022). Alternative splicing diversifies the transcriptome and proteome of the rice blast fungus during host infection. RNA Biol. 19, 373–385. doi: 10.1080/15476286.2022.2043040 35311472 PMC8942408

[B20] Kabbage M. Yarden O. Dickman M. B. (2015). Pathogenic attributes of sclerotinia sclerotiorum: Switching from a biotrophic to necrotrophic lifestyle. Plant Sci. 233, 53–60. doi: 10.1016/j.plantsci.2014.12.018 25711813

[B21] Krogh A. Larsson B. Von Heijne G. Sonnhammer E. L. L. (2001). Predicting transmembrane protein topology with a hidden Markov model: Application to complete genomes. J. Mol. Biol. 305, 567–580. doi: 10.1006/jmbi.2000.4315 11152613

[B22] Livak K. J. Schmittgen T. D. (2001). Analysis of relative gene expression data using real-time quantitative PCR and the 2(T)(-delta delta c) method. Methods 25, 402–408. doi: 10.1006/meth.2001.1262 11846609

[B23] Lyu X. L. Shen C. C. Fu Y. P. Xie J. T. Jiang D. H. Li G. Q.. (2016). A small secreted virulence-related protein is essential for the necrotrophic interactions of sclerotinia sclerotiorum with its host plants. PloS Pathog. 12, e1005435. doi: 10.1371/journal.ppat.1005435 26828434 PMC4735494

[B24] Matera A. G. Wang Z. F. (2014). A day in the life of the spliceosome. Nat Rev Mol Cell Biol 15, 108–121. doi: 10.1186/s13578-017-0153-7 24452469 PMC4060434

[B25] Nickless A. Bailis J. M. You Z. S. (2017). Control of gene expression through the nonsense-mediated RNA decay pathway. Cell Bioscience 7, 26. doi: 10.1186/s13578-017-0153-7 28533900 PMC5437625

[B26] Palusa S. G. Reddy A. S. N. (2015). Differential recruitment of splice variants from SR pre-mRNAs to polysomes during development and in response to stresses. Plant Cell Physiol. 56, 421–427. doi: 10.1093/pcp/pcv010 25637375

[B27] Pan Q. Shai O. Lee L. J. Frey B. J. Blencowe B. J. (2009). Deep surveying of alternative splicing complexity in the human transcriptome by high-throughput sequencing (vol 40, pg 1413, 2008). Nat. Genet. 41, 762–762. doi: 10.1038/ng0609-762d 18978789

[B28] Papasaikas P. Tejedor J. R. Vigevani L. Valcarcel J. (2015). Functional splicing network reveals extensive regulatory potential of the core spliceosomal machinery. Mol. Cell 57, 7–22. doi: 10.1016/j.molcel.2014.10.030 25482510

[B29] Peng Q. Xie Q. X. Chen F. Zhou X. Y. Zhang W. Zhang J. F.. (2017). Transcriptome analysis of sclerotinia sclerotiorum at different infection stages on brassica napus. Curr. Microbiol. 74, 1237–1245. doi: 10.1007/s00284-017-1309-8 28785831

[B30] Pistoni M. Ghigna C. Gabellini D. (2010). Alternative splicing and muscular dystrophy. RNA Biol. 7, 441–452. doi: 10.4161/rna.7.4.12258 20603608 PMC3568746

[B31] Reddy A. S. N. Marquez Y. Kalyna M. Barta A. (2013). Complexity of the alternative splicing landscape in plants. Plant Cell 25, 3657–3683. doi: 10.1105/tpc.113.117523 24179125 PMC3877793

[B32] Shen S. H. Park J. W. Lu Z. X. Lin L. Henry M. D. Wu Y. N.. (2014). rMATS: Robust and flexible detection of differential alternative splicing from replicate RNA-seq data. Proc. Natl. Acad. Sci. United States America 111, E5593–E5601. doi: 10.1073/pnas.1419161111 PMC4280593 25480548

[B33] Si J. R. Pei Y. Shen D. Y. Ji P. Y. Xu R. F. Xue X.. (2021). Phytophthora sojae leucine-rich repeat receptor-like kinases: diverse and essential roles in development and pathogenicity. Iscience 24, 102725. doi: 10.1016/j.isci.2021.102725 34258557 PMC8254037

[B34] Stepankiw N. Raghavan M. Fogarty E. A. Grimson A. Pleiss J. A. (2015). Widespread alternative and aberrant splicing revealed by lariat sequencing. Nucleic Acids Res. 43, 8488–8501. doi: 10.1093/nar/gkv763 26261211 PMC4787815

[B35] Syed N. H. Kalyna M. Marquez Y. Barta A. Brown J. W. (2012). Alternative splicing in plants–coming of age. Trends Plant Sci. 17, 616–623. doi: 10.1016/j.tplants.2012.06.001 22743067 PMC3466422

[B36] Szklarczyk D. Gable A. L. Nastou K. C. Lyon D. Kirsch R. Pyysalo S.. (2021). The STRING database in 2021: customizable protein-protein networks, and functional characterization of user-uploaded gene/measurement sets. Nucleic Acids Res. 49, D605–D612. doi: 10.1093/nar/gkab835 33237311 PMC7779004

[B37] Tollervey J. R. Wang Z. Hortobagyi T. Witten J. T. Zarnack K. Kayikci M.. (2011). Analysis of alternative splicing associated with aging and neurodegeneration in the human brain. Genome Res. 21, 1572–1582. doi: 10.1101/gr.122226.111 21846794 PMC3202275

[B38] Trapnell C. Pachter L. Salzberg S. L. (2009). TopHat: discovering splice junctions with RNA-seq. Bioinformatics 25, 1105–1111. doi: 10.1093/bioinformatics/btp120 19289445 PMC2672628

[B39] Trapnell C. Williams B. A. Pertea G. Mortazavi A. Kwan G. Van Baren M. J.. (2010). Transcript assembly and quantification by RNA-seq reveals unannotated transcripts and isoform switching during cell differentiation. Nat. Biotechnol. 28, 511–515. doi: 10.1038/nbt.1621 20436464 PMC3146043

[B40] Turunen J. J. Niemela E. H. Verma B. Frilander M. J. (2013). The significant other: splicing by the minor spliceosome. Wiley Interdiscip Rev. RNA 4, 61–76. doi: 10.1002/wrna.1141 23074130 PMC3584512

[B41] Wang Z. Gerstein M. Snyder M. (2009). RNA-Seq: a revolutionary tool for transcriptomics. Nat. Rev. Genet. 10, 57–63. doi: 10.1038/nrg2484 19015660 PMC2949280

[B42] Wang E. T. Sandberg R. Luo S. J. Khrebtukova I. Zhang L. Mayr C.. (2008). Alternative isoform regulation in human tissue transcriptomes. Nature 456, 470–476. doi: 10.1038/nature07509 18978772 PMC2593745

[B43] Wang G. H. Sun P. Gong Z. W. Gu L. F. Lou Y. Fang W. Q.. (2018). Srk1 kinase, a SR protein-specific kinase, is important for sexual reproduction, plant infection and pre-mRNA processing in fusarium graminearum. Environ. Microbiol. 20, 3261–3277. doi: 10.1111/1462-2920.14299 30051568

[B44] Wang L. Yang T. Wang B. Lin Q. Zhu S. Li C.. (2020). RALF1-FERONIA complex affects splicing dynamics to modulate stress responses and growth in plants. Sci. Adv. 6, eaaz1622. doi: 10.1126/sciadv.aaz1622 32671204 PMC7314565

[B45] Westrick N. M. Ranjan A. Jain S. Grau C. R. Smith D. L. Kabbage M. (2019). Gene regulation of sclerotinia sclerotiorum during infection of glycine max: on the road to pathogenesis. BMC Genomics 20, 157. doi: 10.1186/s12864-019-5517-4 30808300 PMC6390599

[B46] Williams B. Kabbage M. Kim H. J. Britt R. Dickman M. B. (2011). Tipping the balance: Sclerotinia sclerotiorum secreted oxalic acid suppresses host defenses by manipulating the host redox environment. PloS Pathog. 7, e1002107. doi: 10.1371/journal.ppat.1002107 21738471 PMC3128121

[B47] Will C. L. Luhrmann R. (2011). Spliceosome structure and function. Cold Spring Harb. Perspect. Biol. 3. doi: 10.1101/cshperspect.a003707 PMC3119917 21441581

[B48] Xiao X. Q. Xie J. T. Cheng J. S. Li G. Q. Yi X. H. Jiang D. H.. (2014). Novel secretory protein ss-Caf1 of the plant-pathogenic fungus sclerotinia sclerotiorum is required for host penetration and normal sclerotial development. Mol. Plant-Microbe Interact. 27, 40–55. doi: 10.1094/MPMI-05-13-0145-R 24299212

[B49] Xu B. J. Gong X. Chen S. Hu M. L. Zhang J. F. Peng Q. (2021). Transcriptome analysis reveals the complex molecular mechanisms of brassica napus-sclerotinia sclerotiorum interactions. Front. Plant Sci. 12. doi: 10.3389/fpls.2021.716935 PMC8531588 34691098

[B50] Xu L. S. Li G. Q. Jiang D. H. Chen W. D. (2018). Sclerotinia sclerotiorum: An evaluation of virulence theories. Annu. Rev. Phytopathol. 56, 311–338. doi: 10.1146/annurev-phyto-080417-050052 29958073

[B51] Yang G. G. Tang L. G. Gong Y. D. Xie J. T. Fu Y. P. Jiang D. H.. (2018). A cerato-platanin protein SsCP1 targets plant PR1 and contributes to virulence of sclerotinia sclerotiorum. New Phytol. 217, 739–755. doi: 10.1111/nph.14842 29076546

[B52] Yan Q. Q. Xia X. Sun Z. F. Fang Y. D. (2017). Depletion of arabidopsis SC35 and SC35-like serine/arginine-rich proteins affects the transcription and splicing of a subset of genes. PloS Genet. 13, e1006663. doi: 10.1371/journal.pgen.1006663 28273088 PMC5362245

[B53] Zhang R. X. Calixto C. P. G. Marquez Y. Venhuizen P. Tzioutziou N. A. Guo W. B.. (2017a). A high quality arabidopsis transcriptome for accurate transcript-level analysis of alternative splicing. Nucleic Acids Res. 45, 5061–5073. doi: 10.1093/nar/gkx267 28402429 PMC5435985

[B54] Zhang J. Cui W. Y. Haseeb H. Guo W. (2020a). VdNop12, containing two tandem RNA recognition motif domains, is a crucial factor for pathogenicity and cold adaption inVerticillium dahliae. Environ. Microbiol. 22, 5387–5401. doi: 10.1111/1462-2920.15268 33000558

[B55] Zhang Y. M. Dai Y. F. Huang Y. Wang K. Lu P. Xu H. F.. (2020b). The SR-protein FgSrp2 regulates vegetative growth, sexual reproduction and pre-mRNA processing by interacting with FgSrp1 in fusarium graminearum. Curr. Genet. 66, 607–619. doi: 10.1007/s00294-020-01054-2 32040734

[B56] Zhang Y. M. Gao X. L. Sun M. L. Liu H. Q. Xu J. R. (2017b). The FgSRP1 SR-protein gene is important for plant infection and pre-mRNA processing in fusarium graminearum. Environ. Microbiol. 19, 4065–4079. doi: 10.1111/1462-2920.13844 28654215

[B57] Zhang S. Zhou J. T. Hu H. L. Gong H. P. Chen L. G. Cheng C.. (2016). A deep learning framework for modeling structural features of RNA-binding protein targets. Nucleic Acids Res. 44, e32. doi: 10.1093/nar/gkv1025 26467480 PMC4770198

[B58] Zhu W. J. Wei W. Fu Y. P. Cheng J. S. Xie J. T. Li G. Q.. (2013). A secretory protein of necrotrophic fungus sclerotinia sclerotiorum that suppresses host resistance. PloS One 8, e53901. doi: 10.1371/journal.pone.0053901 23342034 PMC3544710

