# Supplementary figures and images for: Alternative splicing reprogramming in fungal pathogen Sclerotinia sclerotiorum at different infection stages on Brassica napus
Source: Front Plant Sci. 2022 Oct 12;13:1008665. doi: 10.3389/fpls.2022.1008665 (PMC9597501; doi:10.3389/fpls.2022.1008665)

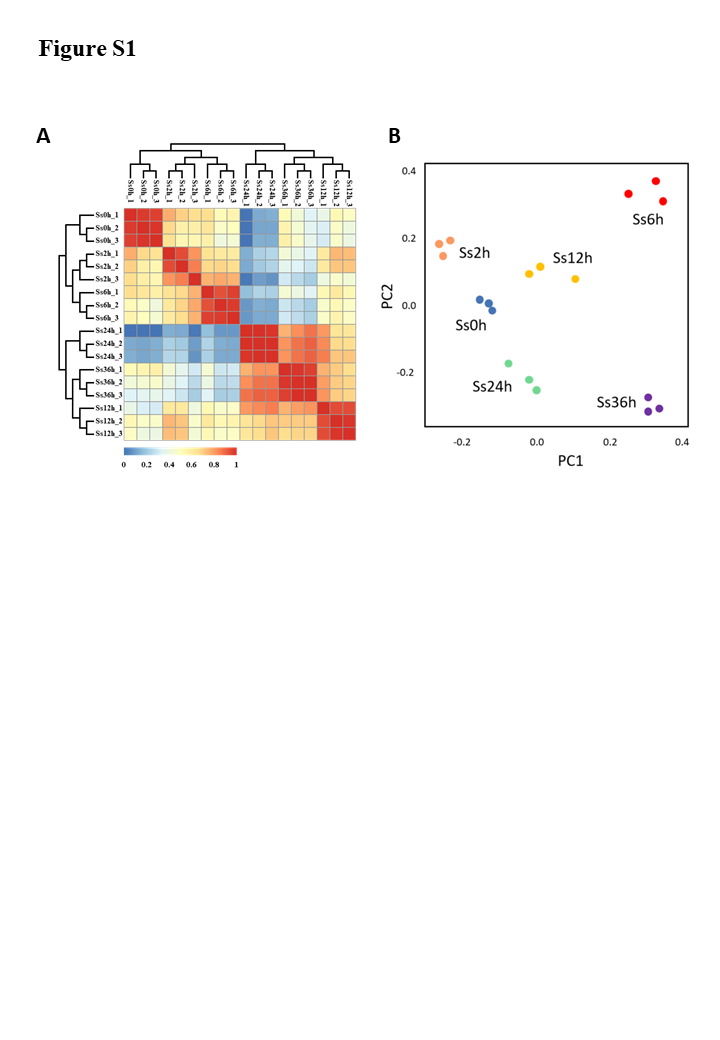

Supplement: Supplementary Figure 1 — Correlation between the RNA-seq data. (A) Hierarchical clustering analysis of the gene expression levels in S. sclerotiorum at 0, 2, 6, 12, 24, and 36 hpi. The color bar represents the distance between samples, with red signifying a short distance and blue signifying a long distance between samples. The color bar (from blue to red) refers to the distance metric used for clustering, with red indicating the maximum correlation value and blue representing the minimum correlation value. (B) Principal component analysis of the gene expression levels in S. sclerotiorum at different infection stages. Each treatment has three independent biological replicates. [file Image_1.tif]

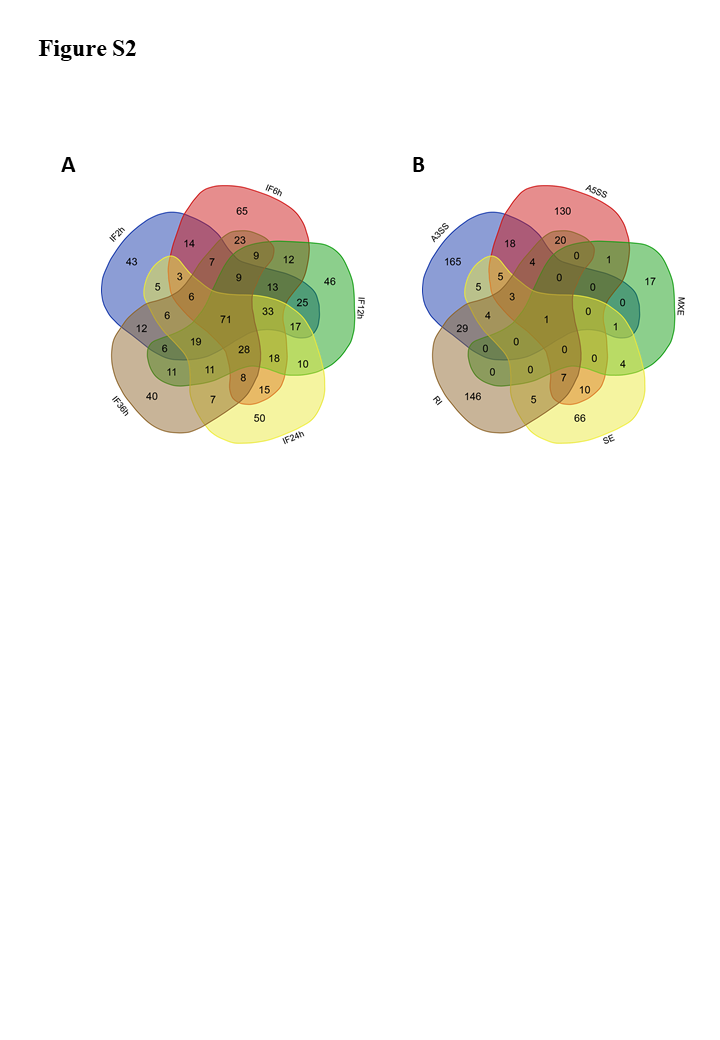

Supplement: Supplementary Figure 2 — Venn diagram of differential alternative splicing genes (DASGs). (A) Venn diagram of DASGs at different infection stages. (B) Venn diagram of DASGs under five alternative splicing types. A3SS, alternative 3′ splice site; A5SS, alternative 5′ splice site; ES, exon skipping; RI, retained intron; MXE, mutually exclusive exon. [file Image_2.tif]

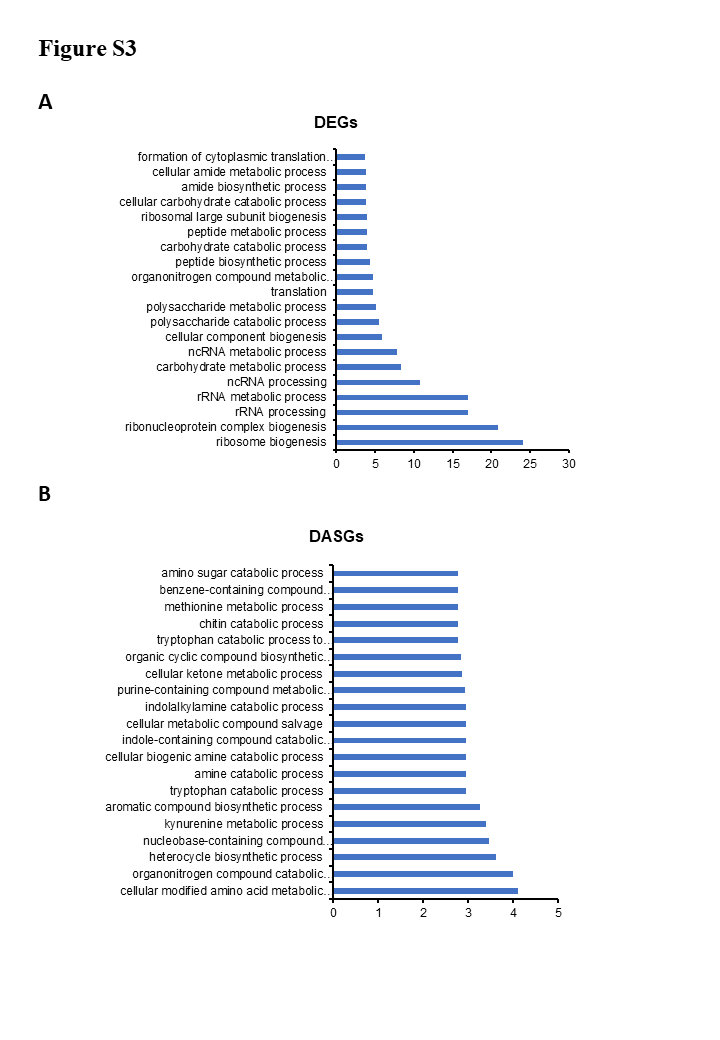

Supplement: Supplementary Figure 3 — Top 20 Gene Ontology (GO) enrichment biological processes of differentially expressed genes and differential alternative splicing genes. The x-axis represents the negative log10 of the P-values for each GO term, and the y-axis indicates the GO terms. [file Image_3.tif]

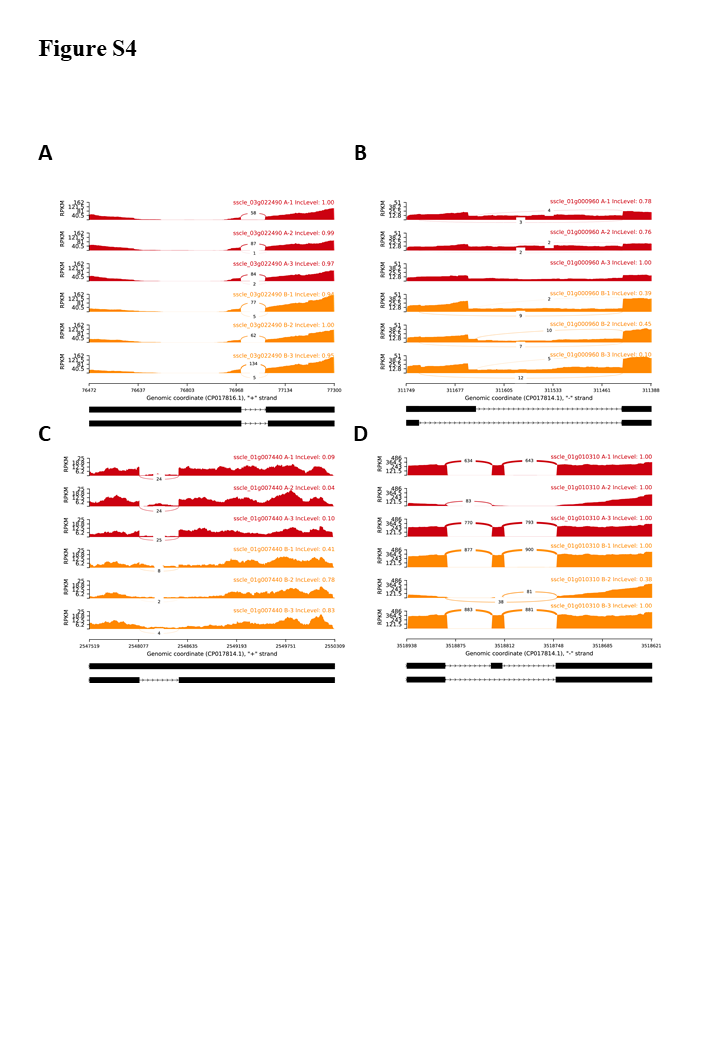

Supplement: Supplementary Figure 4 — Sashimi plots for differential alternative splicing genes (DASGs) in each alternative splicing type. The plots indicate the average RNA-seq read density and splice junction counts for each DASG. The x-axis indicates genomic coordinates, and the y-axis indicates per-base expression. The numbers in the figure show the count of reads across this junction or in this area. The bottom track represents the exon–intron structure (exons in black and introns as lines) of alternative isoforms. (A) Sashimi plot of gene Sscle_03g022490 with A3SS event. (B) Sashimi plot of gene Sscle_01g000960 with A5SS event. (C) Sashimi plot of gene Sscle_01g010310 with SE event. (D) Sashimi plot of gene Sscle_01g007440 with RI event. [file Image_4.tif]

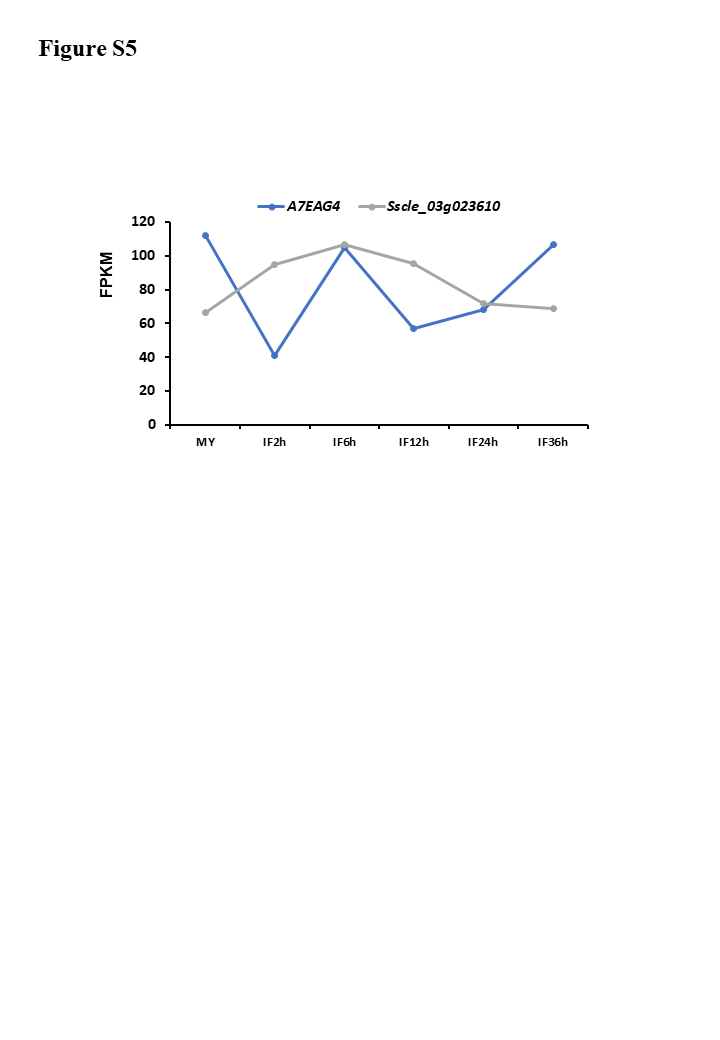

Supplement: Supplementary Figure 5 — Expression patterns of two hub genes in the STRING network. The x-axis represents the infection stages. The y-axis represents the fragments per kilobase of transcript sequence per million base pairs mapped reads value of the transcriptome. [file Image_5.tif]
